# Chinese English as Foreign Language Teachers’ Self-Efficacy and Motivation as Predictors of Burnout

**DOI:** 10.3389/fpsyg.2022.899687

**Published:** 2022-05-30

**Authors:** Meiling Song

**Affiliations:** Logistics and E-Commerce College, Zhejiang Wanli University, Ningbo, China

**Keywords:** EFL teacher, teachers’ self-efficacy, teachers’ motivation, burnout, EFL

## Abstract

In contemporary education, educators are a central and focal component of academic structures accountable for education growth. Educators have an effective role in presenting and planning an effective and important academic program. However, they usually experience burnout due to their stressful job that affects the procedure of their teaching. So, considering the variables that help them in mitigating their burnout should be taken into account. The concept such as educator efficacy has recently attracted significant attention in education fields which by itself emphasizes educators and gets them into the focus of attention in education. Also, the concept of motivation has a strong relationship with the expert aspect of the educators and its significance for language educators and their students are increasingly gaining respect in various worldwide settings, in which lower educators’ motivation is generally a reason for distress. To focus on the predictability role of these constructs on teachers’ burnout, 428 female and male Chinese English as Foreign Language (EFL) teachers from more than 17 cities in nine provinces of China were asked to fill out the three scales, namely, teacher motivation, efficacy, and burnout. The main results of the study, achieved through Pearson Correlation, postulate that the relationships exist among the variables of the study, and by running multiple regression analysis, it is revealed that both variables, namely, self-efficacy and motivation, were the predictors of teachers’ burnout, while self-efficacy was a better predictor. Regarding the applications, educator training plans can focus more on educator self-efficacy and motivation, because of their proven important role in decreasing educator burnout.

## Introduction

Recently, educators are considered the most crucial element for the success of academic structure and attaining favorable results from students and attain a great degree of learners’ educational success because educators significantly affect learners’ learning within the class and school (Sokolov, 2017). Because of the several emotional roles of educators inside the academic field, their psychological wellbeing is considered to have the most importance, as it influences the emotional status of the class which instead affects the educational experience of learners (Vesely et al., 2013). Teaching tension is an element that is harmful to educators’ psychological wellbeing and health (Harmsen et al., 2019). Based on the studies, instruction is one of the careers with the greatest level of work tension, and numerous educators, especially novices, give up their tasks due to work pressures (Newberry and Allsop, 2017; Pishghadam et al., 2019). Tension pertaining to work is associated with dissatisfaction with work, affective burnout, lower work involvement, and instruction inefficacy (Skaalvik and Skaalvik, 2016; Newberry and Allsop, 2017). Tension might also lead to harmful outcomes for educators as well as the instruction quality. Potential outcomes of educator tension are decreased level of satisfaction with career, decreased degree of dedication, higher burnout degrees, and higher educator wearing down (Skaalvik and Skaalvik, 2011; Klassen et al., 2013). Career burnout means the mood wherein people experience bodily and psychological fatigue after high-stress working which is considered as a symptom of emotional fatigue normally seen among people engaged in assisting careers (Shih et al., 2013; Peng et al., 2014). Burnout might be usually seen in careers presenting human services and education as one of those careers (Watts, 2013). In comparison to different careers, burnout in education is most experienced and therefore leads to tension, and speeds up educator’s burnout (Çetin, 2016). Educators’ burnout is described as emotions of no power in an attempt to train learners and build a desirable atmosphere in school for them, no passion to make lessons ready, trouble in encouraging themselves to do a task, lack of power and memory, and disinterest regarding the topic (Seifalain and Derakhshan, 2018). Educators require holding positive emotions and great degrees of motivation pertaining to their career for the schools to achieve their targets (Gün, 2017), and teaching is a crucial component of nurturing a prosperous generation. Thus, well-equipped and prompted educators are highly required within schools. But, studies display an agitating excessive number of educators struggling with burnout difficulties worldwide (Fernet et al., 2016).

The notion of motivation might be a significant basic structure in the procedure of burnout since educators with no motivation have trouble with stressing the identity of education career, inadequate self-efficacy, the deterrence of independence, and insufficient job mechanisms (Dörnyei and Ushioda, 2013). Motivation is a complex part of the human mind and conduct that affects how people wish to use their time, the amount of power they put on each given task, the manner of their thinking and feeling about the activity, and the time they spend on their work (Urdan and Schoenfelder, 2006). Educator’s motivation is a vital notion in each organization, particularly in school. Motivated educators have higher productivity within the academic process by directly affecting the educators’ job efficiency with students and collaborating with other schools and coworkers (Kotherja, 2013). Educators’ motivation has a significant role in learning tasks, educators cannot be forced to educate and obtain the preferred purposes without motivation. Motivation is described as a powerful enhancement that started the attempt to decide the path, severity, and perseverance (Colquitt et al., 2015). Research indicates that learners are susceptible to higher motivation for learning if they understand that their educators are passionate about the class (Lazarides et al., 2018). Accordingly, burnout symptoms, namely, cynical reactions, apprehension, or absence of expert growth, may lead to decreased learner motivation, which weakens education effectiveness and productivity in the long term in an indirect way.

Moreover, recently, scholars are increasingly using the theory of self-efficacy to research job burnout besides investigating the effect of self-efficacy on career burnout formation (Consiglio et al., 2013). Self-efficacy means thinking and judging whether people can complete a task (Kamen et al., 2013). Educator self-efficacy is a vital subject in psychoeducational studies, because of its strong relationship with a vast set of educational variables, motivation, and the educational outcomes of learners within various stages, modalities, and curricular fields of the academic structure (Duffin et al., 2012). Educators’ self-efficacy means motivational structures related to the future indicating educators’ opinions for instruction activities, that is, it specifies how educators consider themselves capable to influence learners’ performance (Bandura, 2010). Educators with excessive rates of class tension had low degrees of self-efficacy in instructional tactics and learner involvement. Moreover, educators’ self-efficacy opinions had a mediating effect on the relationships between class and work tension on career satisfaction (Han and Wang, 2021). Educators feeling efficient within the class will have higher satisfaction in their career and can stay longer in the area and can provide greater strength, creativity, and innovation to their class due to their career consent. Clarifying the procedure of teaching working educators can significantly assist them to have higher motivation in a class environment and enjoy lower burnout (Viel-Ruma Aguayo et al., 2011). Educators can potentially maintain their passion and perseverance and have high flexibility because of their firmer self-efficacy opinions (Tschannen-Moran and Hoy, 2007; Gibbs and Powell, 2012).

Based on the review of literature, academic researchers began to focus on burnout and numerous studies were conducted which confirmed that burnout is an essential issue in the education career and educators are human service employees specifically vulnerable to experience burnout (Johnson et al., 2012; Lauermann and Konig, 2016). In addition, based on a large body of research on the self-efficacy of educators and burnout, numerous scholars hold that burnout and self-efficacy significantly affect the educators’ performance and learners’ learning (Savas et al., 2014; Ghazalbash and Afghari, 2016). Regarding such investigation, there has been much attention to research on the relationship among several educator associated variables, namely, educator self-efficacy, burnout, emotional intelligence, dedication, flexibility, and career consent (Skaalvik and Skaalvik, 2017; Fathi and Derakhshan, 2019; Razmjoo and Ayoobiyan, 2019).

Furthermore, a large body of studies was conducted emphasizing educators’ motivation as an emotional element in the academic context (Claeys, 2011; Paulick et al., 2013). It is generally accepted that the motivation of teachers should be regarded as the most important determining factor of learning upshots. Nonetheless, little empirical evidence exists on the effect of teaching motivation regarding burnout experience among language educators, generally, and EFL educators, particularly (Dörnyei and Ushioda, 2013). Undoubtedly, burnout is referred to as one of the crucial difficulties in lowering the educators’ efficiency; nevertheless, within the Chinese setting, this problem has been given less attention. Therefore, this study aimed to fill this gap and investigate the relationship between self-efficacy motivation and teachers’ burnout among Chinese EFL teachers.

## Review of the Related Literature

### Motivation

Since a long time ago, constructing motivation is regarded as an important aspect of achievement and advancement for humans in their individual and educational lives which is an incentive for any tasks individuals do with which everything gets impossible (Gopalan et al., 2018). Educators’ motivation has a significant function in the learners’ achievement in ESL classes and it refers to the passion and preparation to do things without having instruction or being forced to perform it (Azar and Tanggaraju, 2020), adding that motivation is the procedure of ordering and construing inputs to build a world concept of knowledge. Motivation is classified into seven several intellects, namely, spatial, logical-mathematic, linguistic, musical, physical kinesthetic, and individual intelligence encompassing interpersonal and intrapersonal intelligence (Gardner, 2011).

In the theory of achievement goal (Butler, 2012), educators’ motivation is related to their wishes to gain targets to be successful in their careers. Considering the educators’ self-efficacy (Tschannen-Moran and Hoy, 2007), it is stated that their opinions regarding their capability affect their learners’ success, while motivation affects the attempts they put in education, their perseverance, and conduct. The theory of expectancy-value concentrates on the expectations of people for achievement in an assignment and its understood value, which will forecast their attempt and perseverance in it (Watt and Richardson, 2008). In another theory called self-determination theory (Ryan and Deci, 2020), possibly the most famous theoretical frame for investigating educators’ motivation, educators with greater internalized motivation for their work, or those who get intrinsic delight from it will potentially make investments in their work and indicate flexibility when facing difficulties in their jobs, and instead, they possibly enhance independent motivation in their learners. Motivation falls into two kinds, including internal and external motivation. Internal motivation is described as the natural tendency of people toward learning and integrating. However, external motivation takes place when an activity is performed to obtain something separate. Educators are stimulated to engage in instructing through uniqueness, attentiveness, joy, or gaining academic educational goals and that of their own (Deci and Ryan, 2016). Educators with intrinsic motivation will not want prizes or motivation to begin or finish an activity, while those with external motivation try to achieve acknowledgment or to keep away from being punished (Harun et al., 2019).

### Teacher Efficacy

According to the theory of social cognitive, educators’ self-efficacy is described as educators’ judgment about their abilities to cause preferred results in learner involvement and learning, even among the learners who might be challenging or unmotivated (Tschannen-Moran and Hoy, 2007). A typical educator self-efficacy conceptualization refers to educators’ confidence in their capability to affect valued learner results (Wheatley, 2005). A similar definition is “Single educators’ opinion in their capacity to design, arrange, and conduct tasks needed to obtain intended instructional purposes” (Skaalvik and Skaalvik, 2007). Based on the theory of social cognition, self-efficacy affects individuals’ cognition, feelings, and conduct. As an example, studies on educators indicated that self-efficacy has a positive relationship with career satisfaction and involvement and has a negative relationship with burnout and motivation to quit the education career (Skaalvik and Skaalvik, 2007). Within the past 20 years, some research showed that educator efficacy can be considerably affected by educators’ ideas in their particular instruction context, evaluations of the sources and the accessible aid to them, and the requirements of their instruction activities (Tschannen-Moran and Hoy, 2007).

In his study, Bandura investigated the origin of self-efficiency emergence wherein people as dynamic entities could adjust themselves and change their conduct, instead of being passive entities controlled through unknown protecting powers or internal actions. They can actively participate in altering themselves and manage incidences and phenomena through their measures. Along with Bandura (2010), self-efficacy enhances people’s incentive and cognition sources, which is also a component in coping with particular events. Trust in your self-efficacy forms the basis of motivation, a better living, and personal fulfillment in the entire areas of life (Simarasl et al., 2010). As stated by Bandura (2010), four mechanisms shape self-efficacy, the first of which is that experience or active achievement determines growth in the self-efficacy perception due to achievement in special activities which enhances self-efficacy. An experience or model that is vicarious means a growth of the self-efficacy by watching different people’s success in specific activities. The feeling of “If they can do it, I may also be able to do it” positively affects efficacy. Enhancing efficacy through oral persuasion takes place through encouraging people that they can achieve success in doing the undertaken task. Eventually, physiological elements, namely, becoming highly pressured in challenging conditions, can leave destructive impacts on efficacy (Bandura, 2010). Self-efficacy has several crucial outcomes for people. While considering being able to fulfill an activity may lead to the sensation of pleasure from the task, lower efficacy can cause negative feelings, namely, tension and apprehension, and such emotions can negatively or positively lower the efficiency of people (Prilleltensky et al., 2016). Moreover, this is while those with higher efficacy are buoyant and have higher motivation in tough conditions, and those having lower efficacy may simply quit (Robbins and Judge, 2013).

### Teachers’ Burnout

The burnout construct generally means the syndrome pertaining to work coming from people’s ideas of the main interval among possibilities and perspective of triumphant function and an evident and far less desirable reality (Schaufeli and Taris, 2005). It typically occurs among those whose work requires face-to-face communication, associated with the need for help such as instruction. Burnout comprised three aspect-associated factors such as emotional fatigue, personality loss, and decreased personal achievement (Maslach and Leiter, 2016). While those notions are expanded to educator education, they experience emotional fatigue while they might be effectively emptied in connecting to people, particularly with their learners (Jennett et al., 2003). Affective fatigue features low power and continual exhaustion which is a coral burnout aspect and comes from lengthy tension pertaining to work. The concept of burnout generally refers to a syndrome including emotional fatigue, personality loss, and decreased personal achievement.

A feeling of personality loss takes place when the educator has unconstructive and wrong behavior in relation to others, and unsuitable personal success is faced while educators’ professional productivity and capacity are fatigued (Maslach and Leiter, 2016). A person’s feeling of having lower benefit and capability in their career is considered weak personal effectiveness and it refers to an unconstructive assessment of their career presentation and the overall value of their career (Leiter et al., 2014). Emotional exhaustion underlies the vital fundamentals of burnout and people’s feeling of affective gap due to career tension, conflicts, discomforts, and career overload referring to emotional fatigue (Skaalvik and Skaalvik, 2016). People can experience fatigue in those conditions and may lack enough power and excitation to manage daily career problems (Fathi et al., 2021). Depersonalization is described as a feeling of reluctance and indifference regarding one’s job and to whom one provides service. People suffering from personality loss tend to regard their job and the people they interact with in a deconstructive manner in the job setting (Maslach and Leiter, 2016).

There are three aspects of educators’ burnout: bodily exhaustion, emotional fatigue, and cognitive weariness (Shirom and Melamed, 2006; Wang and Guan, 2020). Both, bodily exhaustion, emotional fatigue, are the same because both highlight the emotional aspect. For this reason, recent research pays attention to the emotional regulation capability, which indicates the main element of affective intelligence and means the potential to modify the affective states of one’s own and that of others (Brackett et al., 2010). The affective-adjustment capability affects how educators state their affections, control tension, and engage with others, and thus, it correlates with the syndrome of burnout (Brackett et al., 2010). The practical affective-adjustment abilities are both essential in this regard and indicate educators’ opinions about such capabilities, meaning that sentimental adjustment is a component of educators’ self-efficacy.

Based on the review of the literature and the above-mentioned gaps, the following research questions are proposed:

RQ1: Do teachers’ self-efficacy and motivation as a whole significantly predict teachers’ burnout?

RQ2: Do teachers’ self-efficacy significantly predict teachers’ burnout?

RQ3: Do teachers’ motivation significantly predict teachers’ burnout?

RQ4: Which one of the two predictors, that is teachers’ self-efficacy or teachers’ motivation is a better predictor of teachers’ burnout?

## Materials and Methods

### Participants

The sample comprised 428 teachers including both genders (male = 142/33.18%, female = 286/66.82%) with different academic qualifications and years of teaching experience. They were from different colleges and universities in various provinces of China with the majority in Zhejiang province and Hebei province (319/74.5%) and other 17 cities in 9 provinces (109/25.5%). Teachers who took part in this study had teaching experience of 1–3, 4–6, 7–10, 11–15, 16–20, and 20–25 years, and more than 26 years which accounted for 28.5, 13.79, 9.81, 13.08, 15.42, 7.24, and 12.15%, respectively. Consent had been given to them before they participated in this research. All responses were based on their willingness.

### Instruments

The following instruments are used in this study.

#### Teacher’s Burnout Scale

Maslach et al. (1996) developed The Maslach Burnout Inventory (MBI), a 22-item Likert scale, as a scale of educator burnout. The questionnaire comprised three subscales, namely, fatigue (9 items), personality loss (5 items), and achievement (8 items). Greater affective fatigue, personality loss, and decreased personal achievement cause excessive burnout conditions. The coefficient of reliability for the questionnaire was computed using a Cronbach’s alpha which turned out to be 0.82. Exploratory factor analysis which indicated a desirable component construct for the inventory was taken into account in this research. The above three burnout aspects are already verified in factor analytic research.

#### Teacher Efficacy Questionnaire

Using a 26-item inventory based on a 7-point Likert scale from 1 to 7, developed by Tschannen-Moran and Hoy (2001), educator efficacy resources were measured. Distinctive aspects of educator efficacy were indicated through three secondary scales within the TSES, namely, educational tactics, class control, and learner involvement. In this study, the internal consistency of the scale was 0.97, which is satisfactory.

#### Teachers’ Motivation Questionnaire

Dweik and Awajan (2013) designed a 10-item inventory so subjects could respond to the questionnaire. Using a 5-point Likert scale of 1–5 (Poorly Motivated to Strongly Motivated), educators have to assign their motivation scores to find out to what extent English language teachers are motivated by the motivational sources. In this study, the scale’s estimated Cronbach’s alpha reliability coefficient was 0.92.

### Data Collection Procedures

To collect the data more smoothly and scientifically, the researcher had carefully designed the questionnaire, invited four professors in translation and applied linguistics to translate it into the target language (Chinese), and then checked for any possible mistakes before the questionnaire was distributed to participants *via* Wenjuanxing, an online data-collection program widely used in China. The whole process lasted 23 days, from January 26 to February 17. To make the results more generalized and reliable, the questionnaire was sent out to 428 teachers with different academic qualifications and majors, working in 32 cities in different provinces of China. All participants were informed of their right to withdraw from the study if they felt any discomfort or offense in this study. They were also notified of how to properly fill in the questionnaire with guidance which was provided at the top of the questionnaire. Then, the researcher carefully cleansed and checked the data before it was sent to SPSS for further analysis, and also probe into the research question of the study.

### Data Analysis

Pearson Correlation was implemented to examine the probable relationship among the key variables of this research. Furthermore, a linear multiple regression analysis was used to answer the second research question to check the predictor role of efficacy and motivation on their burnout.

## Results

This study aimed to investigate the role of Chinese EFL teachers’ self-efficacy and motivation as predictors of their burnout. Due to the nature of the study, first, the reliability of the instruments is calculated. Since reliability is sample-dependent, it is deemed necessary to ensure that the instruments of this study had acceptable levels of internal consistency. Therefore, the collected data were analyzed *via* running Cronbach’s alpha. Table 1 displays the results of Cronbach’s alpha and descriptive statistics for the three instruments.

**TABLE 1:** Results of Cronbach’s alpha and descriptive statistics for the three instruments.

	Mean	Std. deviation	Variance	Skewness		Kurtosis			
					
	Statistic	Statistic	Statistic	Statistic	Std. error	Statistic	Std. error	Cronbach’s alpha	No. of items
Teachers’ burnout	70.39	21.90	479.92	0.148	0.118	0.393	0.236	0.827	22
Teachers’ motivation	30.34	12.11	146.86	0.121	0.118	0.283	0.236	0.925	10
Teachers’ self-efficacy	109.62	33.24	1105.19	0.159	0.118	0.116	0.236	0.970	26
Valid N (listwise)									

As presented in Table 1, the corresponding alpha values for the teachers’ burnout, motivation, and self-efficacy turned out to be 0.82, 0.92, and 0.97, respectively, which are all above 0.70 and considered acceptable (Hulin et al., 2001). To answer the research questions, a standard multiple regression was run. Multiple regressions have several assumptions that need to be checked before applying this statistical test. The first assumption is the sample size. Based on Tabachnick and Fidell (2013, p. 123), the sample size should be “*N* > 50 + 8 m (where m = the number of independent variables).” In this study, the number of cases is 428 which is well beyond the required sample size and thus, this assumption is met. The next assumption was multicollinearity which was checked by checking the correlations table.

**FIGURE 1:**
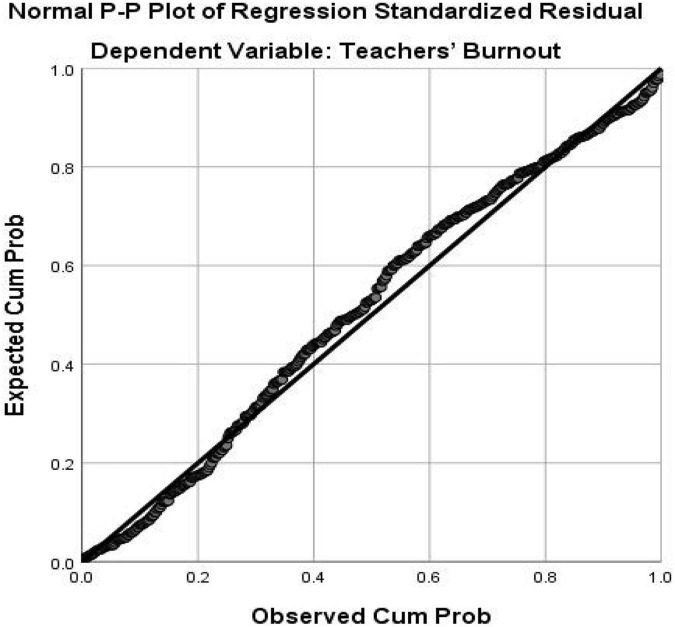
Normal probability plot (P-P) of the regression standardized residual (Teachers’ burnout is the dependent variable).

**FIGURE 2:**
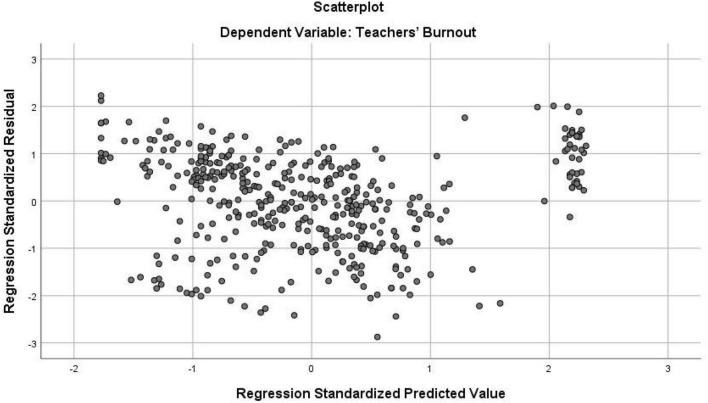
Scatter plot with teachers’ self-efficacy as dependent variable.

As indicated in the above table, the correlation between the two independent (predictor) variables equals 0.527 which is lower than 0.7, and thus the multicollinearity assumption is warranted (Pallant, 2020). To check multicollinearity further, the VIF index was also checked. As seen in Table 2, the VIF indices are not above 10 which is an indication of the lack of violation of this assumption. Normality, linearity, homoscedasticity, and independence of residual assumptions were checked by inspecting the Normal Probability Plot (P-P) of the Regression Standardized Residual and the Scatterplot. Normal Probability Plot (P-P) of the Regression Standardized Residual and the Scatterplot are demonstrated in Figures 1, 2, respectively.

**TABLE 2:** Coefficients for teachers’ self-efficacy and motivation as predictors of burnout.

Model		Unstandardized coefficients		Standardized coefficients	*t*	Sig.	95.0% Confidence interval for B		Correlations			Collinearity statistics	
								
		*B*	Std. error	Beta			Lower bound	Upper bound	Zero-order	Partial	Part	Tolerance	VIF
a	(Constant)	107.190	3.237		33.1	0.000	100.82	113.552					
	Teachers’ motivation	±0.434	0.093	±0.240	±4.68	0.00	±0.616	±0.252	±0.428	±0.222	±0.19	0.669	1.4
	Teachers’ self-efficacy	±0.215	0.034	±0.327	±6.37	0.00	±0.282	±0.149	±0.465	±0.296	±0.26	0.669	1.4

*^a^Dependent Variable: Teachers’ Burnout.*

As depicted in Figure 1, all the dots lie in a diagonal line from the top right to the bottom left which is an indication of normal data for multiple regression analysis (Pallant, 2020).

As presented in Figure 2, points are scattered in a rectangular shape which suggests no violation of linearity, homoscedasticity, and independence of residuals assumptions. Moreover, no cases lie out of the range of ± 3.3 which is an indication of the non-existence of outliers for multiple regression analysis. Upon checking the assumptions, the researcher ran multiple regression analyses. Table 3 shows the model summary and ANOVA results for the multiple regression analysis.

**TABLE 3:** Model summary and ANOVA test of regression analysis.

Model	*R*	*R* ^2^	Adjusted *R*^2^	Std. Error of the estimate	F	df1	1. df2	Sig.
b	0.505^a^	0.225	0.251	18.95	75.23	2	424	0.000

*^a^Predictors: (Constant), Teachers’ Self-efficacy, Teachers’ Motivation.*

*^b^Dependent Variable: Teachers’ Burnout.*

As seen in Table 3, as a whole, teachers’ self-efficacy and motivation explained about 22% of the variance in the dependent variable (teachers’ burnout). In other words, teachers’ self-efficacy and motivation made a 22% contribution to explaining the variance in teachers’ burnout. This amount of contribution was found significant as the *F*-value was significant [*F*(2, 424) = 75.23, *p* = 0.00 < 0.05]. To find which one of the independent variables, that is, teachers’ self-efficacy and teachers’ motivation, was a better predictor for teachers’ burnout, Beta values were checked. Table 2 displays the respective results.

As seen in Table 2, the sig value corresponding to teachers’ motivation equals 0.00 which is lower than 0.05 indicating that teachers’ motivation is a significant predictor of teachers’ burnout. Similarly, the sig value for teachers’ self-efficacy is 0.00 which is less than 0.05 indicating that teachers’ self-efficacy is also a significant predictor of teachers’ burnout. As seen in Table 2, the Beta value corresponding to teachers’ self-efficacy equals −0.327, which is bigger than the Beta value belonging to teachers’ motivation which equals −0.240. As Pallant (2020) contends, Beta values in the coefficients table should be considered irrespective of their negative and positive mathematical signs. Thus, it can be concluded that teachers’ self-efficacy is a better predictor of teachers’ burnout compared to teachers’ motivation. Note should, however, be taken that such prediction should be interpreted inversely since the correlation indices between teachers’ burnout, on one hand, and teachers’ self-efficacy and motivation, on the other hand, are negatively significant as seen in Table 4.

**TABLE 4:** Correlations table of the variables.

		Teachers’ burnout	Teachers’ motivation	Teachers’ self-efficacy
Pearson correlation	Teachers’ burnout	1.000	−0.428	−0.465
	Teachers’ motivation	−0.428	1.000	0.575
	Teachers’ Self-efficacy	−0.465	0.575	1.000
Sig. (1-tailed)	Teachers’ burnout		0.000	0.000
	Teachers’ motivation	0.000		0.000
	Teachers’ self-efficacy	0.000	0.000	
N	Teachers’ burnout	427	427	427
	Teachers’ motivation	427	427	427
	Teachers’ self-efficacy	427	427	427

## Discussion

This study aimed at probing if teachers’ self-efficacy and motivation as a whole significantly predict teachers’ burnout. Moreover, the study set out to investigate if teachers’ self-efficacy significantly predicts teachers’ burnout. In a similar vein, the study sought to explore if teachers’ motivation significantly predicts teachers’ burnout. Finally, the study aimed at examining which one of the two predictors, that is teachers’ self-efficacy or motivation, is a better predictor of teachers’ burnout. The results of multiple regression indicated that teachers’ self-efficacy and motivation as a whole significantly predict teachers’ burnout. Finally, it was shown that teachers’ self-efficacy was a better predictor of teachers’ burnout compared to teachers’ motivation. The results of the study showed that self-efficacy is one of the noteworthy issues in educators’ presentation in the classroom, which should be taken into consideration in the educational cycle. The results are in agreement with the study carried out by Schwarzer and Hallum (2008) and Federici and Skaalvik (2012) who indicated that a negative relationship exists between self-efficacy and depersonalization and emotional exhaustion as components of burnout. Moreover, it is undoubtedly proved that the results of this study are in line with some other inquiries in this domain (Yazdi et al., 2014; Kosevic and Loh, 2015; Fernet et al., 2016; Cansoy et al., 2017). The results are consistent with the results of the study by Ventura et al. (2015), who showed that beliefs regarding professional efficiency significantly correlated with burnout and involvement. Especially, expert self-efficacy had a positive correlation with involvement and it had a negative correlation with burnout.

The results are in line with some inquiries (Durr et al., 2014; Dicke et al., 2015), who concluded that educators having higher individual sources and skills, namely, self-efficacy, compatible managing tactics, and expert knowledge, will potentially overcome the demanding situations of education career and consequently have a lower chance of experiencing burnout. Studies also found that emotional fatigue and personality loss in educators happen because of sustained expert pressure produced as a result of their incapability to successfully control the class. Consequently, there is an inverse association between educators’ burnout and self-efficacy (Skaalvik and Skaalvik, 2007). Brudnik (2009) studied the level of protection of educators’ self-efficacy when faced with burnout emphasizing that self-efficacy kept educators safe from the burnout syndrome components like personality loss, affective fatigue, and decreased self-fulfillment. Indeed, educators develop constructive attitudes toward learners and teaching structures, if they have a high degree of self-efficacy. In contrast, a low degree of self-efficacy is related to anxiety; as a result, it may be concluded that self-efficacy is a managing resource when faced with tension and burnout. Educators with higher efficacy succeed in their careers. They suffer lower career burnout because they are confident in their capabilities to handle stressful and difficult conditions with higher effectiveness, while educators with lower self-efficacy are apprehensive, depressed, prone to affections, and exhausted in terms of emotion as they encounter difficulties. If people believe that they can fulfill a certain challenge, they can do it quite well in comparison to those skeptical or uncertain about their careers (Kreitner and Kinicki, 2007).

Correspondingly, the correlation between teachers’ burnout and their motivation is indirect but significant, which means those students who are not stimulated and motivated enough deal with more burnout in the process of their teaching means that EFL teachers who are less inspired in completing their class assignments may potentially get emotionally exhausted. Motivation improves the efficiency of people within the work if their fundamental mental requirements are met (Deci and Ryan, 2016). Therefore, second language educators’ motivation to teach must be improved and their mental requirements must be met such that they might function well in second language classes. The results are in line with the results by Cox (2017) who believed that teacher motivation has a significant function in avoiding burnout and they declared that it can be regarded as the most actual clarification to the difficulties of stress, burnout, and unhappiness.

Based on the literature, educators’ motivation will improve performance within the class and, as a result, enhance schooling and the school structure quality. The quality of educational learning possibilities may just be increased by qualified and dedicated educators (Watt and Richardson, 2012). Educators directly affect learners’ motivation to learn in any dimension of their experience in class, and learners mostly react positively to a well-structured class instructed by a passionate educator (Khamis et al., 2008). It could be said that the factor of motivation enhances the educators’ success, along with the educational skill and experience in school, meaning that educators who are motivated indicate great educational performance without losing a positive mindset about education and school despite a boring school environment that correspondingly can diminish or lesson their burnout level. If educators get effectively exhausted, they will not actively and passionately get engaged in managing the class time and developing assignments and tasks, and failing to make investments in power and innovation in their efforts might normally reflect itself within the class and devalue their viewpoints, efforts, and motivation. Another study on the same line showed that a strong and negative relationship exists between self-efficacy and burnout level, meaning that the lower burnout, higher the self-efficacy (Bümen, 2010).

## Conclusion and Implications

The research has several practical implications for educators of EFL, educator trainers, educator training developers at the college, and educators generally. This research assists practitioners and educators in EFL teaching areas to expand their comprehension of the importance of efficacy and motivation and their effect on burnout. Regarding the considerable effect of educator self-efficacy in decreasing instruction pressure, EFL educator training developers ought to take the required measures to improve EFL educators’ feeling of efficacy such that they can successfully manage stressful conditions that result in less burnout. Therefore, the English Language education society can move to expertise, where educators are inspired to gain an expert identity which itself helps to enhance self-efficacy and motivation, and decrease burnout among educators.

Educator training plans have to focus more on educator self-efficacy and motivation because of their proven important role in decreasing educator burnout. In such a viewpoint, EFL educator trainers are suggested to take realistic measures to nurture educators’ motivation and self-efficacy to assist pre-service and in-service educators in coping with demanding conditions with higher efficiency. Therefore, educator training plans have to concentrate on educators’ expert identity which has a strong relationship with educators’ motivation as well as self-efficacy. Educators must nurture their efficacy because while the educator is confronted with a demanding activity, great self-efficacy provides a feeling of peace and confidence to shape the motive to acquire achievement. The higher belief of educators will lead to higher work motivation to let them develop.

Besides, the educator is concerned with controlling the affective atmosphere of the class, fostering a constructive feeling among the learners, and preferably instructing with exhilaration, passion, and interest (Dewaele et al., 2018). One can use the results to reform in-service teaching classes, that is, such classes may be redesigned in various methods, which nurtures and improves educators’ self-efficacy opinions. Policy-makers of foreign language and beneficiaries ought to put enough attempts to provide educators with an environment full of comfort and free from threatening in the second language educational settings as well as providing more freedom and independence to EFL educators to assist them to obtain more self-efficacy and understanding in the class atmosphere.

It is suggested that language institutes and schools help improve their educators’ self-efficacy through constructing a helpful environment, giving authority and sufficient freedom to the educators, and growing a feeling of attachment among employees. A plan centered on enhancing class control abilities will also be useful based on research that found that such education leads to better reports regarding health and motivation, such as a decrease in emotional exhaustion.

Because the outcomes of the research showed that educators’ self-efficacy was a considerable variable predicting burnout, the ones suffering from burnout must be aided to enhance their self-efficacy to obtain their belief in their ability. Using several qualitative studies, techniques like observing or interviewing are suggested for helping an in-depth understanding of the burnout–self-efficacy relationship. The administrative personnel of the school has to have a look at the educators showing the burnout symptoms such that they could offer them expert help to handle burnout. In the meanwhile, if administrative personnel hold teaching sessions on burnout and the methods to struggle with it, then it can be beneficial.

In this research, the educators’ teaching experience and their personality types are not taken into consideration. More studies can be done to consider these issues in their inquiries, as well. Various researchers are suggested to redo this research, both qualitatively and quantitatively, to investigate the differences among findings throughout those variables. Moreover, future researchers can improve the generalizability of such findings by using qualitative or mixed approach study designs to triangulate the results, as such research can potentially offer a deeper comprehension. Furthermore, the research may be conducted again to discover different variables, namely, age, socio-cultural characteristics, and academic level, within the demographic data. The research may be conducted again to study the same variables inside the settings apart from EFL settings.

## Data Availability Statement

The original contributions presented in the study are included in the article/supplementary material, further inquiries can be directed to the corresponding author.

## Ethics Statement

The studies involving human participants were reviewed and approved by the Zhejiang Wanli University Academic Ethics Committee. The patients/participants provided their written informed consent to participate in this study.

## Author Contributions

The author confirms being the sole contributor of this work and has approved it for publication.

## Conflict of Interest

The author declares that the research was conducted in the absence of any commercial or financial relationships that could be construed as a potential conflict of interest.

## Publisher’s Note

All claims expressed in this article are solely those of the authors and do not necessarily represent those of their affiliated organizations, or those of the publisher, the editors and the reviewers. Any product that may be evaluated in this article, or claim that may be made by its manufacturer, is not guaranteed or endorsed by the publisher.
